# Long Term Survivals in Aggressive Primary Brain Malignancies Treated With an Adjuvant Ketogenic Diet

**DOI:** 10.3389/fnut.2022.770796

**Published:** 2022-05-03

**Authors:** Kenneth A. Schwartz, Mary Noel, Michele Nikolai, Lawrence K. Olson, Norman G. Hord, Micheal Zakem, Justin Clark, Mohamed Elnabtity, Bryan Figueroa, Howard T. Chang

**Affiliations:** ^1^Colleges of Human and Osteopathic Medicine, Michigan State University, East Lansing, MI, United States; ^2^Department of Medicine, College of Human Medicine, Michigan State University, East Lansing, MI, United States; ^3^College of Osteopathic Medicine, Michigan State University, East Lansing, MI, United States; ^4^Department of Family Practice, College of Human Medicine, Michigan State University, East Lansing, MI, United States; ^5^Department of Food Science and Human Nutrition, College of Agriculture and Natural Resources, Michigan State University, East Lansing, MI, United States; ^6^Department of Physiology, College of Natural Sciences, Michigan State University, East Lansing, MI, United States; ^7^Department of Nutritional Sciences, Harold Hamm Diabetes Center, College of Allied Health, University of Oklahoma, Oklahoma City, OK, United States; ^8^College of Human Medicine, Michigan State University, East Lansing, MI, United States

**Keywords:** glioblastoma multiforme, ketogenic diet, diet therapy, long term survival, verified ketosis

## Abstract

Aggressive primary brain tumors (APBT) glioblastoma multiforme and grade IV astrocytoma are treated with multimodality treatments that include surgery to remove as much tumor as possible without sacrificing neurological function followed by radiation therapy and chemotherapy usually temozolomide. Survivals in adults are in the range of 8–16 months. The addition of a ketogenic diet (KD) to rodents with transplanted brain tumors increased survival in nine of 11 animals to over 299 days compared to survival in untreated controls of 33 days and radiation only controls of 38 days. We treated humans with APBT with standard of care neurosurgery immediately followed by 6 weeks of an adjuvant ketogenic diet concurrent with radiation therapy and temozolomide. Twice daily measurements of blood ketones and glucose were recorded and the patients' diet was modified toward the goal of maintaining blood ketone levels approaching 3 mM. Of the nine patients who completed the protocol three younger patients age 32, 28, and 22 at enrollment are alive and employed with clinically stable disease and brain images 74, 58, and 52 months since diagnosis. All the six older patients mean age 55 have died with disease progression detected on average 8 months after Dx. In conclusion: 1. It is possible to implement and maintain dietary induced ketosis in patients with APBT; 2. The longer survivals observed in younger patients treated with KD need to be confirmed in larger studies that should be focused on younger patients possibly under age 40.

## Introduction

While treatments for many malignancies have advanced to more targeted therapies, systemic treatments for aggressive primary brain malignancies (glioblastoma multiforme, GBM, also known as WHO grade IV astrocytoma) continue to rely on alkylating agents that can cross the blood brain barrier (usually temozolomide, TMZ) (1). Current therapy includes surgical resection of as much of the tumor as possible without impairing vital brain functions, followed by radiation therapy and TMZ (2). The addition of TMZ prolongs survival on average 2.5 months (3). Bevacizumab (Avastin®) does not increase survival in patients with newly diagnosed GBM, but is helpful in the treatment of relapsed patients (4). Wearing an “electric hat” for alternating electric field therapy is reported to prolong survival on average by 2.7 months (5). These therapies prolong life incrementally with median survivals in adult patients in the range of 8–16 months (6).

Studies including rodent models suggest that adding a ketogenic diet (KD) to some of the standard treatments used in humans may prolong survival. The addition of KD to radiation therapy markedly prolonged the life of nine of 11 rodents. Histological evaluation 299 days post GBM implantation showed that nine of 11 animals were free of disease. This compared with survival of 33 days in untreated controls, and 38 days in animals treated with just radiation (7). Reports from other investigators also demonstrate increased survival in animals treated with KD, with retarded growth of their brain malignancy (8–11).

We report long term follow up of nine adult patients with aggressive primary brain tumors who, following their initial neurosurgery, were treated with 6 weeks of an adjuvant KD combined with standard of care radiation therapy and chemotherapy with TMZ. The patients' blood ketones were measured twice daily and the results were used to make adjustments in their diets to assure that ketosis was maintained during the entire 6 weeks of the study period.

## Methods

### Clinical Protocol

After signing informed consent 12 patients were enrolled in our clinical trial protocol that was IRB approved (11-452s). Two patients (#1 and #2, **Tables 1, 2**) were studied with the original protocol that stipulated starting the KD after they failed conventional treatments, and 10 were treated with the revised protocol that started the KD at the same time as the initial radiation and chemotherapy treatments, and continued KD for 6 weeks. This protocol had the primary objectives of investigating side effects attributable to the KD, as well as noting tumor response and time to progression. Patient #3 withdrew before completing just 4 weeks of the diet because he had to return to work as a long haul truck driver and could not complete the protocol after the fourth week. Nine patients completed the revised protocol.

The inclusion criteria were participants must be over 16 years of age, had histologically confirmed diagnosis of GBM, had an Eastern Cooperative Oncology Group performance status of ≤ 2, a life expectancy of >3 months, could tolerate a high-fat diet, and had the ability to give informed consent. The exclusion criteria were participants may not have diabetes mellitus, may not have had a cholecystectomy within a year prior to entering the study, did not have any malignancy other than the brain cancer, had not participated in another investigational study within 2 weeks prior to this study's entrance, did not have brain metastasis from a non-brain primary tumor, did not have any major comorbidity such as liver, kidney, or heart failure, and were not pregnant.

The protocol KD was caloric balanced, based on the patient's starting weight and was constructed using the KetoDietCalculator software program so that the ratio in grams of fat to combined grams of protein and carbohydrates was 3:1. The calculation ranges used for all subjects in the original protocol was 20–25 kilocalories (kcal)/kilogram (kg) of body weight, considered a mild restriction. The protein was low at 20 kcal/kg body weight so 25 kcal/kg was used to provide minimum of 0.6 gram (g) protein (pro)/kg. Actual meals plan for all subjects started as a range of 23–25 kcal/kg. In that range, with a 3:1 ratio, a 0.6–0.7 g pro/kg was provided. Calculations for an 1,800 kcal daily intake yields the following range of macronutrients: 1,566 fat kilocalories (174 grams of fat) 234 protein and carbohydrate kilocalories (58.5 grams). Protein grams for the meal plan are based on the subject's weight to achieve at least 0.6 gm pro/kg and the remaining kcal from carbohydrates. A gram food scale was given to each participant to ensure the correct measurements of each food item as calculated by the KetoDietCalculator. Other studies have used 1,600 kcal and higher fat ratio of 4:1 but those provide even lower protein so are not sustainable for muscle maintenance.

Before starting and after completing the KD protocol, the patients had a history and physical exam along with complete blood counts, chemistries, lipids, and uric acid. During the 6 weeks of KD, the patients recorded their daily weights, and twice daily measurements of blood glucose and ketones obtained upon waking prior to eating and evenings 2 h after eating. Each patient was given an Omron Model HBF-400 Scale for their daily weights, an Abbot Precision Xtra Meter with test strips to measure their blood ketones and glucose twice daily, a log to record their results and a food scale. Participants received dietary instruction by a registered dietitian who developed a meal plan and menus for each subject. In addition, a dietitian called or visited the patients regularly (at least once a week) to review results of the patients' glucose and ketone measurements as they related to the food logs which were kept by the patients throughout their time on the KD. Follow up examination and imaging were at the discretion of the referring physician.

### Protocol Revision, Tolerability, and Side Effects

Our initial protocol stipulated starting of the KD after tumor growth was demonstrated following the patients' initial treatment with surgery, radiation and temozolomide. We revised our protocol based on a report, in a rodent model, that nine of 11 mice with a transplanted primary brain tumor that were treated with a KD simultaneously with radiation therapy survived, whereas all of the control mice and mice treated with only radiation therapy or only KD died (7). The success of this simultaneous dual treatment in animals prompted a revision of our protocol so that patients' initial post-surgery treatment included a KD begun at the same time as radiation therapy and chemotherapy. The enrolled patients maintained ketosis for 6 weeks with support from their family and/or caregivers and our dieticians (MN, and MMN). Participants maintained blood glucoses under 100 mg/dl and blood ketones around 1–2 mM. All participants lost weight, averaging about 5 lbs. Combining the KD with standard-of-care radiation and chemotherapies did not add any significant side effects to the patients' therapy.

## Results

**Figure 1:**
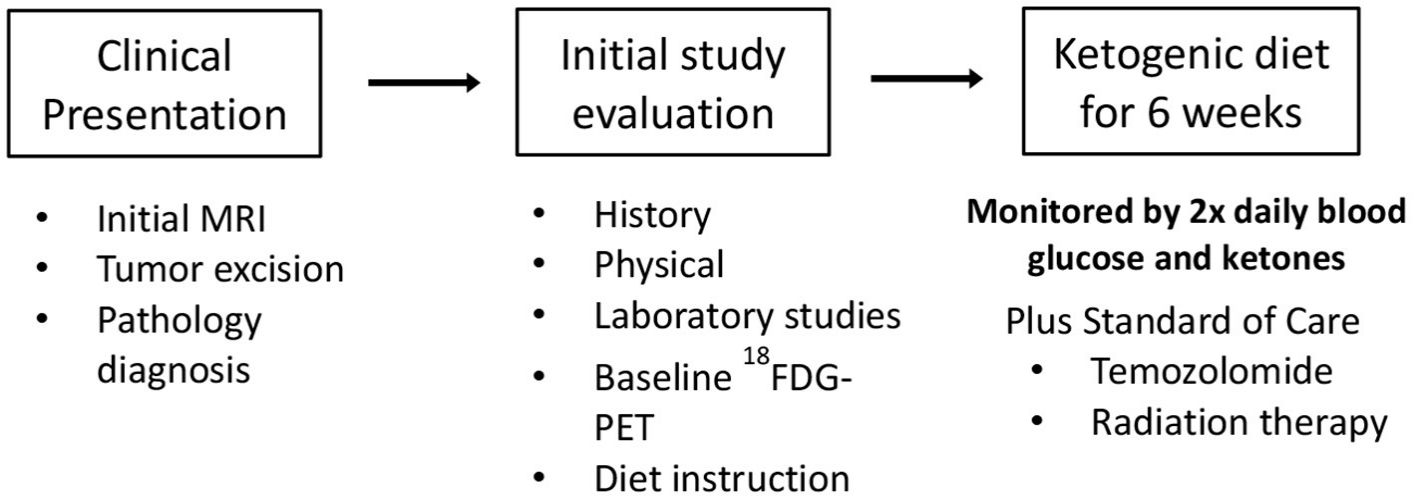
Schematic of the ketogenic diet protocol.

**Figure 2:**
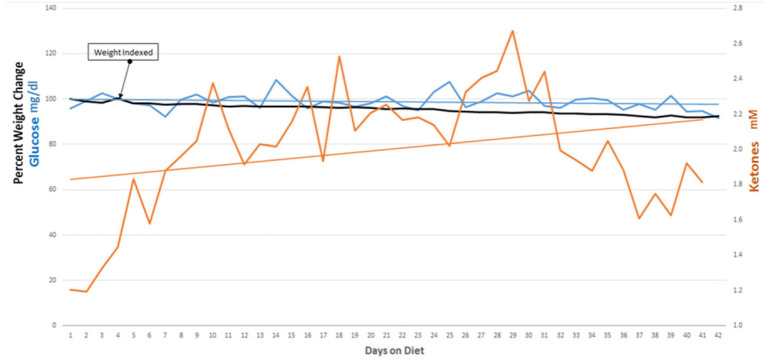
Daily glucose and ketone averages and weight change.

Nine of 10 patients completed the revised protocol (Figure 1) with KD therapy initiated at the same time that they started their treatments with radiation therapy and TMZ. Patient #3 had to return to work as a long haul truck driver and could not complete the protocol after the fourth week. Blood glucose and ketone concentrations were measured twice daily, fasting in the morning when the patients first awoke and in the evening before they went to bed. Table 1 shows the means and standard deviations for each patient's ketone and glucose measurements on days 1 and 14 and averages throughout the study. Aggregate averages for all the patients and lines of linear best fit are depicted in Figure 2 for the patients' twice daily blood glucose and ketone measurements as well as their daily weights.

**Table 1:** Blood glucose and ketones: first and last days and averages.

**Averages glucose**	**Through day 42**								
	**First glucose**			**Last glucose**			**Ave-glucose**		
**Pt#-Age-Gen**	**Ave** **±StdDev**			**Ave** **±StdDev**			**Ave** **±StdDev**		
1 55 M	90	±	11.1	80.2	±	8.3	85.6	±	11
2 52 M	81.7	±	15	81.7	±	7	81.7	±	11.7
3 71 M	106.8	±	13.7	123.5	±	26.2	114.4	±	21.8
4 32 M	97.9	±	9.3	100.2	±	11.1	98.9	±	10.1
5 57 M	102.6	±	10.7	104.5	±	8.1	103.5	±	9.5
6 35 M	116.5	±	23.5	115.9	±	25.9	116.2	±	24.4
7 37 M	88.9	±	8.8	99.7	±	12	94.2	±	11.8
8 67 M	82.1	±	11	90	±	10.6	85.9	±	11.4
9 28 F	87.1	±	10.2	84.1	±	8.5	85.6	±	9.4
10 22 M	80.4	±	9.8	92	±	15.1	85.3	±	13.4
11 68 M	137.2	±	20.2	146	±	30.5	141.5	±	25.9
12 63 M	97.5	±	8	107.5	±	11.7	102.5	±	11.2
**Average ketones**	**Through day 42**								
	**First ketone**			**Last ketone**			**Ave- ketone**		
**Pt#-Age-Gen**	**Ave** ***±*****StdDev**			**Ave** ***±*****StdDev**			**Ave** ***±*****StdDev**		
1 55 M	2.7	±	0.7	4.1	±	0.7	3.3	±	1
2 52 M	2.9	±	0.9	4	±	1.1	3.5	±	1.1
3 71 M	1.5	±	0.9	2.6	±	1.1	2	±	1.1
4 32 M	1.6	±	1.2	1.8	±	0.8	1.7	±	1
5 57 M	2.1	±	0.5	2.8	±	0.7	2.5	±	0.7
6 35 M	0.9	±	0.7	2	±	1.1	1.4	±	1.1
7 37 M	0.7	±	0.4	2	±	0.9	1.3	±	0.9
8 67 M	1.1	±	0.6	1.2	±	0.6	1.1	±	0.6
9 28 F	3	±	0.8	3.5	±	0.9	3.3	±	0.9
10 22 M	1.5	±	1.1	2.6	±	1.4	2	±	1.3
11 68 M	0.4	±	0.2	1.2	±	0.7	0.8	±	0.6
12 63 M	0.6	±	0.3	1.5	±	0.6	1	±	0.7

Patient outcomes for the 12 patients initiating the KD protocols are presented in Table 2. The two patients (Patients # 1 and 2) who were treated with KD after they had progression of their disease following initial standard of care had died as a result of their GBM. Patient #3 failed to complete the required 6 weeks of the protocol. Nine patients (#4 thru 12) completed the protocol and were treated with the KD simultaneously with standard of care radiation and chemotherapies administered following their neurosurgery. Three of these nine patients are progression-free 80, 64, and 62 months since diagnosis. These three long term survivors were younger: 32, 28, and 22 years old at the time of diagnosis. The other six patients were older, mean age 55 at diagnosis, and had progression of their disease 8, 11, 9, 6, 6, and 6 months after their diagnosis. The two patients who are alive 80 and 62 months without progression had GBM that were positive for the isocitrate dehydrogenase-1 R132H mutation (IDH1-R132H). The tumor of the third long-term progression-free patient was grade III astrocytoma (IDH mutation status undetermined).

**Table 2:** Patient outcomes.

**Original protocol:**				
**Treated with KD after tumor progression on standard of care treatment**				
**Pt#**	**Age**	**Sex**		
1	55	M	Tumor progression on ketogenic diet	
2	52	M	Tumor progression on ketogenic diet	
**Revised protocol:**				
**Treated with KD at same time as standard of care treatment**				
			**Months to progression**	**Months of progression free survival**
3*	71	M		
4	32	M		**80**
5	57	M	8	
6	35	M	11	
7	37	M	9	
8	67	M	6	
9	28	F		**62**
10	22	M		**64**
11	68	M	6	
12	63	M	6	

* *Patient 3 withdrew before completing just 4 weeks of the diet. The bold values indicates patients alive without disease progression*.

## Discussion

Younger patients with GBM are reported to have a better prognosis (12–17). A recent report documents long term survival in a 26 year old male treated for GBM with only a KD who is alive 80 months post diagnosis with just slow growth of this tumor documented by serial imaging studies (18). Our present study showed three of nine patients treated with an adjuvant ketogenic diet along with standard treatments of surgery and radiation are still alive 80, 62, and 64 months from diagnosis with no evidence of disease advancement. These long-term survivors were all younger (age 32, 28, and 22) when diagnosed as compared to those who died with progression of disease (mean age 55 at diagnosis).

Longer survival in younger patients suggests that aggressive primary brain cancers in these patients may have a biological propensity for slower growth and/or a greater sensitivity to treatment with a ketogenic diet along with radiation and chemotherapy (2, 19). Tumors of two of our patients tested positive for the IDH 131/132 polymorphism associated with longer survival (20). These features at diagnosis suggested that these patients may have had a favorable prognosis. Methylation of the MGMT promoter (21) was not evaluated in our study. The six older patients treated in our study, mean age 55, did not live longer than expected and probably did not benefit from the addition of the ketogenic diet to their therapy. Our results align with previous reports showing that age is a critical factor for survival (22, 23). We suggest that future studies evaluating the addition of a ketogenic diet to GBM therapy should be targeted toward younger patients perhaps 40 years of age or younger (24).

After their initial neurosurgery, radiation, chemotherapy and adjuvant ketogenic diet treatments, all of our patients reported some decrease in mental capacity. One of the surviving patients prior to his GBM diagnosis worked as an investment counselor. After his initial surgeries, radiation and chemotherapy, he could not perform the executive functions quick enough to continue working in that capacity. However, he is able to work in a position that does not require such a high level of executive function. All three of the surviving patients have returned to full employment.

The cooperation of the patients, their families and caregivers was essential for preparation of the foods in the ketogenic diet and twice-a-day monitoring and recording of blood glucose and ketone levels. Following neurosurgery, patients may require assistance to check their blood ketones and implement changes in their diet based on their ketone concentrations. It is critical that the patients' ketotic state be verified with twice daily checks of the level of ketones in their blood. Ongoing reinforcement of the protocol was provided by dietitians (MMN or MN) who helped to maintain the patients' ketotic state throughout the 6 weeks study. If needed, dietary modifications were made by the dietitian (MN) to keep the twice daily blood ketone levels approaching 3 mM. Patients and their families agreed that 6 weeks was about as long as they could adhere to the dietary specifications and restrictions stipulated by the ketogenic diet.

It was hypothesized that aggressive primary brain tumors may not be able to metabolize ketones like normal brain tissues depriving the tumor tissue of nutrients required for survival and growth and this was the rationale used to initiate this study (25). However, ketone metabolism in human brain tumors does not differ from metabolism in neighboring normal brain, suggesting that selective ketone metabolic differences between normal and malignant brain cells may not be a plausible mechanism for the proposed antineoplastic effects of dietary induced ketosis (26). β-hydroxybutyrate is the main ketone produced with a ketogenic diet and is known to function as a histone deacetylase inhibitor which affects translation of DNA and this may be part of the mechanism responsible for the significant anti-tumor effects observed in controlled studies using animal models of aggressive primary brain cancers treated with a ketogenic diet (8, 9, 26–29).

Long term survival with aggressive primary brain cancer is possible (30–33). The three younger patients reported here are alive and working with stable brain images and clinical exams following treatment with standard of care neurosurgery followed by radiation, temozolomide and an adjuvant ketogenic diet. The diet was implemented and adjusted over time by registered dieticians experienced with using the ketogenic diet for patients with intractable seizures (34). Following neurosurgery our patients needed help from family members for executive functions and this included assistance with food preparation and adjustments to their diets based on the results of twice a day measurements of blood glucose and ketones.

Previous studies demonstrated that it is possible to prescribe a ketogenic diet in patients with primary aggressive brain malignancies (35–38). Our study extends these reports by enlisting the cooperation of family members and dietitians to help insure maintenance of the ketotic state. Whether the diet contributed to the longevity in our three surviving patients is a question that can only be answered by larger studies. Since 12 older patients with verified diet induced ketosis, six in our study and six reported by van der Leuw (24) did not appear to benefit from an adjuvant ketogenic diet, we suggest that future evaluations of the ketogenic diet in patients with aggressive primary brain cancer be restricted to younger patients, possibly under 40 years of age.

## Data Availability Statement

The original contributions presented in the study are included in the article/supplementary material, further inquiries can be directed to the corresponding author.

## Ethics Statement

The studies involving human participants were reviewed and approved by Institutional Review Board (IRB), Michigan State University. The patients/participants provided their written informed consent to participate in this study.

## Author Contributions

All authors listed have made a substantial, direct, and intellectual contribution to the work and approved it for publication.

## Funding

We remain grateful for initial funding through a grant from Michigan State University College of Human Medicine, and a grant from the VFWRN031117(15SCH) and a grant from The American Institute for Cancer Research #207193.

## Conflict of Interest

The authors declare that the research was conducted in the absence of any commercial or financial relationships that could be construed as a potential conflict of interest.

## Publisher's Note

All claims expressed in this article are solely those of the authors and do not necessarily represent those of their affiliated organizations, or those of the publisher, the editors and the reviewers. Any product that may be evaluated in this article, or claim that may be made by its manufacturer, is not guaranteed or endorsed by the publisher.
